# Functional Connectivity in Tactile Object Discrimination—A Principal Component Analysis of an Event Related fMRI-Study

**DOI:** 10.1371/journal.pone.0003831

**Published:** 2008-12-02

**Authors:** Susanne Hartmann, John H. Missimer, Cornelia Stoeckel, Eugenio Abela, Jon Shah, Rüdiger J. Seitz, Bruno J. Weder

**Affiliations:** 1 Department of Neurology, Kantonsspital St. Gallen, St. Gallen, Switzerland; 2 Paul Scherrer Institute, PSI, Biomolecular Research, Villigen, Switzerland; 3 fMRIB Centre, Department of Clinical Neurology, University of Oxford, Oxford, United Kingdom; 4 Department of Neurology, University Hospital Düsseldorf, Düsseldorf, Germany; 5 Brain Imaging Centre West, Research Centre Jülich, Jülich, Germany; 6 University of Berne, Berne, Switzerland; Lund University, Sweden

## Abstract

**Background:**

Tactile object discrimination is an essential human skill that relies on functional connectivity between the neural substrates of motor, somatosensory and supramodal areas. From a theoretical point of view, such distributed networks elude categorical analysis because subtraction methods are univariate. Thus, the aim of this study was to identify the neural networks involved in somatosensory object discrimination using a voxel-based principal component analysis (PCA) of event-related functional magnetic resonance images.

**Methodology/Principal Findings:**

Seven healthy, right-handed subjects aged between 22 and 44 years were required to discriminate with their dominant hand the length differences between otherwise identical parallelepipeds in a two-alternative forced-choice paradigm. Of the 34 principal components retained for analysis according to the ‘bootstrapped’ Kaiser-Guttman criterion, t-tests applied to the subject-condition expression coefficients showed significant mean differences between the object presentation and inter-stimulus phases in PC 1, 3, 26 and 32. Specifically, PC 1 reflected object exploration or manipulation, PC 3 somatosensory and short-term memory processes. PC 26 evinced the perception that certain parallelepipeds could not be distinguished, while PC 32 emerged in those choices when they could be. Among the cerebral regions evident in the PCs are the left posterior parietal lobe and premotor cortex in PC 1, the left superior parietal lobule (SPL) and the right cuneus in PC 3, the medial frontal and orbitofrontal cortex bilaterally in PC 26, and the right intraparietal sulcus, anterior SPL and dorsolateral prefrontal cortex in PC 32.

**Conclusions/Significance:**

The analysis provides evidence for the concerted action of large-scale cortico-subcortical networks mediating tactile object discrimination. Parallel to activity in nodes processing object-related impulses we found activity in key cerebral regions responsible for subjective assessment and validation.

## Introduction

Tactile exploration, an acquired skill learned early in childhood, constitutes the basis for tactile object recognition and somatosensory discrimination. During action-related somatosensory information processing, the fingers explore with directed motion, adapting exactly to the objects [1]. The kinetic signals transmitted by the spindle apparatus of the muscles and joints convey the size as well as the three dimensional characteristics of the explored object. Action-related and perception-related somatosensory processing most probably take segregated routes, the former terminating within the posterior parietal lobe and the latter projecting through somatosensory area II (SII) to the insula [2]–[4]. The superior parietal lobule (SPL) and the adjacent intraparietal sulcus are critically involved in specific processing of the perceived kinesthetic cues during action-related somatosensory information processing. While lesions of these areas are associated with tactile apraxia and produce a unimodal sensory deficit with executive and perceptive components [5], [6], activation studies established in humans a fronto-parietal circuit responsible for object manipulation [7], [8].

In the following we treat the somatosensory discrimination of solid parallelepipeds, i.e. blocks of pure aluminum identical in all characteristics: volume, weight, surface texture, etc., with the exception of surface area and length. The task was developed and the basic prerequisites for the stimulation paradigm described in detail by Roland and Mortensen [9]. The parallelepipeds were chosen as objects presenting an elementary aspect of form free of ordinary associations. They served as the stimuli of an activation paradigm in which regional cerebral blood flow (rCBF) was measured as the subjects distinguished between the lengths of a pair of parallelepipeds [1]. Essential features of the paradigm are the restricted duration of the sequential exploration of each object in the pair and of the decision, characterizing a sequential two-alternative forced-choice task, as well as the nonverbal communication of the decision. Manipulation, i.e. sensory-guided motor activity without a cognitive load, was additionally performed and served as reference task.

The present study analyses functional magnetic resonance imaging (fMRI) data obtained during this stimulation paradigm using principal component analysis (PCA). In comparison to categorical analysis describing activation maxima, the network description is of considerable importance, since brain processes do not take place in single cortical areas but involve functional circuits [10]–[18]. PCA evaluates the covariance of all possible voxel pairs, yielding orthogonal spatial patterns and subject-condition expression coefficients that are statistically uncorrelated [19].

The rationale of the present exploratory data analysis was to divide the task into phases characterized by their specific sensorimotor and cognitive aspects. Precondition is a subdivision of the task according to its event-related phases of manipulation, exploration, comparison and discrimination of objects, interval and recovery providing the opportunity to perform inferential statistics among the conditions. Only this formal statistical comparison of task conditions identifies PC images suggesting conclusions with respect to the biological relevance of a pattern of interregional covariance (or PC). We hypothesized that: (1) PCA specifically distinguishes differentiated networks subserving somatosensory object discrimination common to the subjects; (2) these networks will be revealed by decomposing the stimulation paradigm into its phases of manipulation, exploration and recovery and (3) these functional circuits accommodate highly differentiated processes including sensory signal processing, focusing of attention, memory encoding and assessment of acquired information.

## Materials and Methods

### Participants

Seven right-handed males between 22 and 44 years of age participated in the study. Handedness was determined using the Edinburgh Handedness Inventory as modified by Salmaso and Longoni [20]; an average score of +89 indicated the dominance of the right hand in the participants. None of the subjects presented neurological or psychological disorders at the time of the study. All provided their written informed consent prior to the study in conformance with the Declaration of Human Rights (Helsinki 1975). The study was approved by the Ethics Committee of the Heinrich-Heine-University Düsseldorf.

### Stimulation Paradigm

The simulation paradigm consisted of a sequential, two-alternative forced-choice requiring the discrimination of parallelepipeds with respect to their oblongness. Consisting of many repetitions, the paradigm demanded a sustained level of directed attention [21]. Basically, the subjects explored parallelepipeds with their right hand. All objects had identical volume (11.5 cm^3^) and weight (32.5 g) and were made of nonmagnetic, hard aluminum; four different parallelepipeds differing in their oblongness, characterized by the dimensions of the major axes and the square bases, were presented. The difference between the lengths of the major axes of one pair, 3.97 mm, exceeded the discrimination threshold determined in previous studies with a probability of explicit recognition of 95% in normal volunteers; the difference between the lengths of the major axes of the second pair, 0.44 mm, was below the threshold, as indicated by pure guessing in normal volunteers. The square bases of the pairs were indistinguishable [20]. The proportion of pairs with supra- and subthreshold difference in the long axis was balanced. In order to compel attention and to anticipate habituation effects, approximately one fifth of the object pairs were identical; the presentation of identical pairs obliged the subjects to wait for the second parallelepiped before making a decision. Pure manipulation of spheres served as control task for the haptic information processing during exploration of the parallelepipeds.

**Figure 1:**
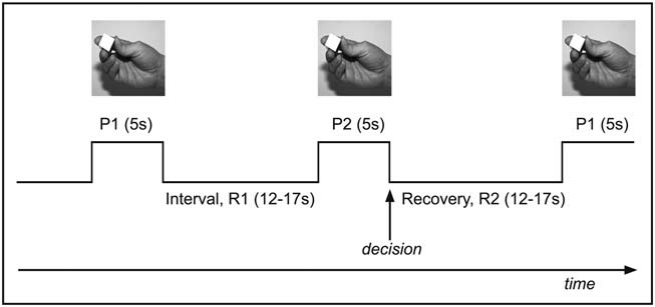
Structure and time course of the study paradigm. Each sequence consisted of four phases: (1) exploration of object 1, P1; (2) the interval R1 during which the shape characteristics of P1 were encoded in working memory; (3) exploration of object P2, on-line comparison of P2 with the encoded characteristics of P1 and the decision; and (4) the pause R2 between sequences.

The forced-choice stimulation paradigm comprised four phases: (1) presentation of the first object for tactile exploration, P1; (2) interval between presentations, i.e. holding the extracted information about object 1 in working-memory, R1; (3) presentation of the second object for tactile exploration accompanied by on-line comparison of this object with the memory of the first object and followed by the decision, P2; (4) interval or recovery before the exploration of the next object pair, R2. Each object was placed in the right hand for five seconds (s); the intervals between presentation of the objects in a pair and after presentation of the second object lasted between 12 and 17 s (Fig. 1). This protocol, distributing the onsets of all conditions stochastically throughout the image acquisition time, provided the same sensitivity of BOLD response for all slices in the image volume.

In a control task, the subjects manipulated spheres of the same volume and weight as the parallelepipeds with their right hand without making a choice; the sequence of active sphere manipulation (Ss) and interval was the same as in the forced-choice paradigm.

The subjects lay supine in the scanner with their heads immobilized and eyes closed. Directed via earphones connected to a computer outside the scanner room, an investigator presented and removed the objects promptly on an acoustic signal. The objects were always placed in the same manner on the palm, the long axis parallel to the thenar. The subjects explored the presented object continuously with the fingers of their right hand. While the subjects only manipulated the identical spheres, they were instructed to choose which member of a pair of parallelepipeds was more oblong. The subjects were requested to extend their right thumb as soon as the object was removed if the second object was estimated to be more oblong, or solely to open the hand for the next object if the first was longer or if they could not detect any difference. Each session consisted of the presentations of 34 pairs of parallelepipeds or 68 spheres; four sessions were acquired for each subject, two each of parallelepipeds and spheres. Subjects did not leave the scanner between sessions. The presentation of the parallelepipeds and spheres was done separately in order to ease the demanding task of the attendant, namely the quick and correct presentation of the objects. Furthermore, this procedure allowed reliable comparison of haptic information processing with the reference task of pure manipulation, i.e. sensory-guided motor activity without a cognitive load. The order of presentations (pairs with suprathreshold or subthreshold differences in the long axis) and the order of parallelepipeds within a pair (more or less oblong) were pseudorandomized within and across sessions, implying that the first and second members of pairs were longer an equal number of times. The order of exploration of parallelepipeds and manipulation of spheres was also pseudo-randomized. Subjects were free to choose an exploration strategy but were asked to keep moving their fingers during the entire 5 s of the exploration phase. To permit off-line analysis of the explorations, the sessions were recorded by a video recorder located outside the scanner room viewing the subject close-up through a window.

Half an hour prior to scanning, the course of the experiment was explained to the subjects in detail, but they did not practice the task before the acquisitions.

### fMRI acquisition

The acquisitions were performed with a Siemens Vision 1.5 T scanner (Erlangen, Germany) using an EPI-GE sequence with a repetition rate (TR) of 5 s, an echo time (TE) of 66 ms and a flip angle of 90°. Covering the whole brain, the image volumes consisted of 30 transaxial slices oriented parallel to the bi-commissural plane with a minimal resolution in plane of 3.125×3.125 mm^2^, a slice width of 4 mm and distance between slices of 0.4 mm. Each session consisted of 255 volumes. The first three volumes of each session were disregarded in the analysis. In addition to the functional images, a high-resolution, anatomical T1-weighed image was acquired for each subject, consisting of 128 sagittal slices with a minimal in-plane resolution of 0.9 mm (TR = 40 ms, TE = 5 ms, flip angle = 40°).

### Data analysis

Image preprocessing and categorical comparisons used modules of the Statistical Parametric Mapping software (SPM99, Wellcome Department of Cognitive Neurology, London, UK; available online at http://www.fil.ion.ucl.ac.uk/spm).

Preprocessing included slice-time correction, realignment, spatial standardization to the standard brain provided by the Montreal Neurology Institute (MNI), and spatial smoothing using a Gaussian filter with an isotropic full width at half maximum (FWHM) of 10×10×10 mm^3^ to accommodate the resolution of the scanner and intersubject variability. The dimensions of the re-sampled images were 79×95×68 voxels and the voxel sizes 2×2×2 mm^3^. The anatomical T1-weighted image of each subject was coregistered to the mean image of the functional images and transformed to the standard MNI space. Realignment parameters as determined in the realignment step were used as confounding covariates. Temporal filtering consisted of a Gaussian low frequency filter of FWHM 4 s and a high frequency filter of FWHM 100 s, as recommended in SPM99. For global normalization, all image volumes were scaled to the overall grand mean. Using the hemodynamic response functions provided by SPM, presentations of the first and second parallelepipeds (P1 and P2) were modeled separately for each subject in a first level analysis, as were the pauses between and following the presentations (R1 and R2), respectively. The four phases of the paradigm were further distinguished in the modeling according to the length differences of the parallelepipeds, i.e. above the threshold (Pa), below the threshold (Pb) or identical (Pid). The presentation of the spheres was also modeled (Ss), but not the pauses in-between. Thus, the procedure yielded 13 regressors, i.e. image volumes related to specific phases of the task, for each of the seven subjects, i.e. P1a, P1b, P1id, R1a, R1b, R1id, P2a, P2b, P2id, R2a, R2b, R2id and Ss. The data sets of each subject for statistical evaluation consisted of 28 repetitions each of P1a, P1b, R1a, R1b, P2a, P2b, R2a, R2b, 12 each of P1id, R1id, P2id, and R2id, and 136 each of Ss. The cerebral coordinates are reported in Talairach space [22]. A freely available Matlab script (http://www.mrcbu.cam.ac.uk/Imaging/mnispace.html) effected the transformation from MNI space [23].

### Principal component analysis

PCA was executed using in-house software of which some modules were adapted from SPM. Extracerebral voxels were excluded from the subject-condition image volumes, using a mask derived from the gray matter component yielded by segmentation of the anatomical image volume into gray matter, white matter and cerebrospinal fluid. Calculation of the residual matrix was the first step. From a matrix whose rows corresponded to the 91 conditions (seven subjects * 13 regressors) and columns to the fifty-five thousand voxels in a single image volume, corresponding to the mask, were subtracted from each element the mean of voxel values of its column and the mean of voxel values of its row and added the grand mean of all voxel values in the original matrix. The result is the residual matrix for which the row, column and grand means vanish. Using the singular value decomposition implemented in Matlab, the residual matrix was then decomposed into 91 components, consisting of an image, an expression coefficient, and an eigenvalue for each component. The procedure differs from that of Alexander and Moeller only in that the data were not transformed logarithmically before computation of the residual matrix [19]. The eigenvalue is proportional to the square root of the fraction of variance described by each component, the expression coefficients describe the amount that each subject and condition contributes to the component, and the component image displays the degree to which the voxels covary in the component. The PCs reflect the variance among both conditions and subjects. The expression coefficients and voxel values of a principal component are orthonormal and range between −1 and +1; the orthogonality reflects the statistical independence of the PCs. The expression coefficients can be subjected to statistical tests, e.g. unpaired t-tests revealing significant mean contrasts between groups of subjects or conditions (“subject-condition expression coefficients”), or tests of regression or correlation with behavioral measures. Note that the term condition in subject-condition expression coefficient includes both the phases P1, R1, P2, R2 and the characterization of length differences above (a) and below (b) threshold, and identical (id).Voxels satisfying selected thresholds indicate the physiological interpretation of the component [10]. In the absence of a statistical theory to evaluate the significance of the PCs, three sets of empirical criteria were applied:

1. To estimate the number of PCs to be retained for analysis, we employed a ‘bootstrapped’ Kaiser-Guttman approach which determines the critical component whose eigenvalue lies closest to the average of all component eigenvalues and retains only those PCs lying within the 95 % confidence limits of the critical component [24].
2. Nine t-tests constituted a minimal set of independent comparisons distinguishing mean contrast patterns among the 13 regressors of the paradigm. This included: i) four comparisons of the phases P1 and P2 with the following recovery for both distinguishable and indistinguishable objects: P1a with R2a, P1b with R2b, P2a with R2a and P2b with R2b (Table 1,1–4), ii) comparison of the second presentations of distinguishable and indistinguishable objects, P2a with P2b, since P2a was assumed to reflect explicit somatosensory discrimination (i.e. the primary aim of the study) (Table 1, 5), iii) two comparisons of the second presentations P2 for distinguishable and indistinguishable objects with manipulation of spheres in order to further assess the differing cognitive load in the two comparisons (Table 1,6–7), iv) comparison of the first and second presentations, P1 with P2, for indistinguishable objects in order to explore frustrated attempts at explicit somatosensory discrimination (Table 1,8), and v) comparison of manipulation of spheres with the following pauses as a baseline for haptic information processing (Table 1,9). Only PCs satisfying the Bonferroni correction at significance threshold of p<0.05 were selected for further analysis.
3. For the interpretation of the images of PCs selected by the t-tests to reflect significant mean contrasts, only those voxels of relevant components for which the voxel values lie in the first (negative load) or ninety-ninth percentile of voxel values (positive load) were analyzed.

### Categorical comparison using SPM99

To establish a relationship of the PCA to conventional analyses, comparable categorical comparisons of the 13 conditions were formulated as t-tests and evaluated in a mixed-effects model using SPM. The image volumes analyzed were the same as in the principal component analysis. The analyses served as a reference for the PCA, relating the voxels covarying the most to those showing maximum intensity differences. The contrasts of the categorical comparisons were therefore chosen to correspond to the sets of conditions that selected relevant principal components. The comparisons corresponding to the principal components 1 and 3 consisted of unpaired, two-tailed t-tests with the 68 and 33 degrees of freedom, respectively, given by the number of conditions. A threshold p<0.001 corrected for multiple comparisons and a minimum cluster size of 16 voxels provided the significance criteria. The comparisons corresponding to principal components 26 and 32 consisted of paired, two-tailed t-tests with 6 degrees of freedom. To account for the few degrees of freedom the threshold, p<0.01 uncorrected for multiple comparisons and a minimum cluster size of 8 voxels provided the significance criteria. The extent threshold corresponds to the mean cluster size expected of a random t-distribution with the same number of degrees of freedom.

## Results

### Behavioral data

We classified finger movements during manipulation and exploration according to Roland and Mortensen into encompassing (very few), rolls (spheres only) and dynamic digital [9]. Rolls and dynamic digital movements involved mainly fingers 1 to 3. Thumb frequency during dynamic digital movements was on average 2 Hz, consistent with earlier observations [1], [20]. The discrimination rate for the rectangular parallelepipeds was in the range predicted from previous observations with a mean of 77% (62–84%, 95% CI) [1], [20]. Subdviding the responses according to the length differences, correct answers were given with a probability of 91% (86–94%, 95% CI) in the case of suprathreshold differences, indicating explicit perception, and with probability of 57% (50–64%, 95% CI) in the case of subthreshold differences, indicating random choice. For identical parallelepipeds correct answers occurred with probability of 63% (53–72%, 95% CI).

### Selection of the principal components

**Figure 2:**
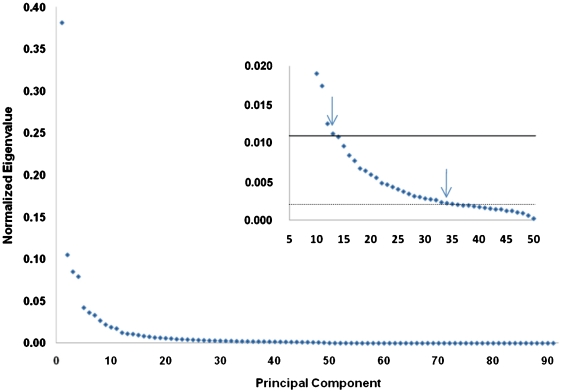
Scree plot showing the fraction of variance accounted for by each PC. The main graph shows discontinuities after the first, fourth and eleventh PC. The insert displays the subspace between the tenth and the fiftieth PC, illustrating the ‘bootstrapped’ Kaiser-Guttman criterion. The solid, upper line shows the average of normalized eigenvalues, defining PC 13 as the critical component (left arrow), and the dashed line the inferior 95% confidence interval which indicates the component retention down to PC 34 (right arrow).

Figure 2 shows the eigenvalue distribution of the 91 principal components normalized to represent the percent of total variance described by each PC. The distribution is marked by discontinuities that suggest subgroups of components: the first principal component (PC 1) accounts for 38% of the variance, PCs 2–4 for almost 27%, PCs 5–11 for 19.7%, and PCs 12–32 for 12.5%. PC 13 is the critical component according to the Kaiser-Guttman criterion, and the lower 95 % confidence limit (‘bootstrapped’ Kaiser-Guttman) indicates that PCs 14–34 be included in the analysis. Four PCs (PC 1, PC 3, PC 26 and PC 32) showed significantly different means between subject-condition expression coefficients corresponding to phases of the paradigm according to unpaired, two-tailed t-tests. For the nine independent t-tests (Table 1) applied to the 34 investigated components, three of the four salient components (PC 1, 3 and 26) yielded t-tests implying probabilities of less than one false positive in the 306 comparisons. The t-test implicating PC 32 yielded a significance corresponding to 1.5 false positives or a Bonferroni corrected significance of p<0.05.

**Table 1:** Unpaired, two-tailed t-tests of subject-condition PC expression coefficients.

Principal component			1	3	26	32
Fraction of Variance [%]			38.2	8.5	0.4	0.3
Cumulative Fraction of Variance [%]			38.2	57.2	95.5	97.2
1. P1a	vs	R2a	0.012	0.022	0.211	0.782
2. P1b	vs	R2b	0.013	0.137	0.009	0.180
3. P2a	vs	R2a	**0.000**	**0.003**	0.151	**0.005**
4. P2b	vs	R2b	**0.000**	**0.001**	0.014	0.158
5. P2a	vs	P2b	0.831	0.578	0.034	0.009
6. P2a	vs	Ss	0.282	0.087	0.432	0.009
7. P2b	vs	Ss	0.456	0.035	0.168	0.571
8. P1b	vs	P2b	0.456	0.118	**0.002**	0.437
9. Ss	vs	R2	**0.000**	0.131	0.390	0.062

Of the 34 PCs admitted to analysis by the Kaiser-Guttman criteria, only PC 1, 3, 26 and 32 showed significant differences (p<0.05 corrected for multiple comparisons according to the Bonferroni t procedure, in bold) in any of the nine indicated t-tests comparing subject-condition expression coefficients corresponding to the phases of the paradigm. P1 and P2, explorations of first and second parallelepipeds; R1, interval between explorations; R2, recovery between presentations of pairs; Ss, exploration of the spheres; a, above and b, below the discrimination threshold.

### Images of the relevant principal components

**Figure 3:**
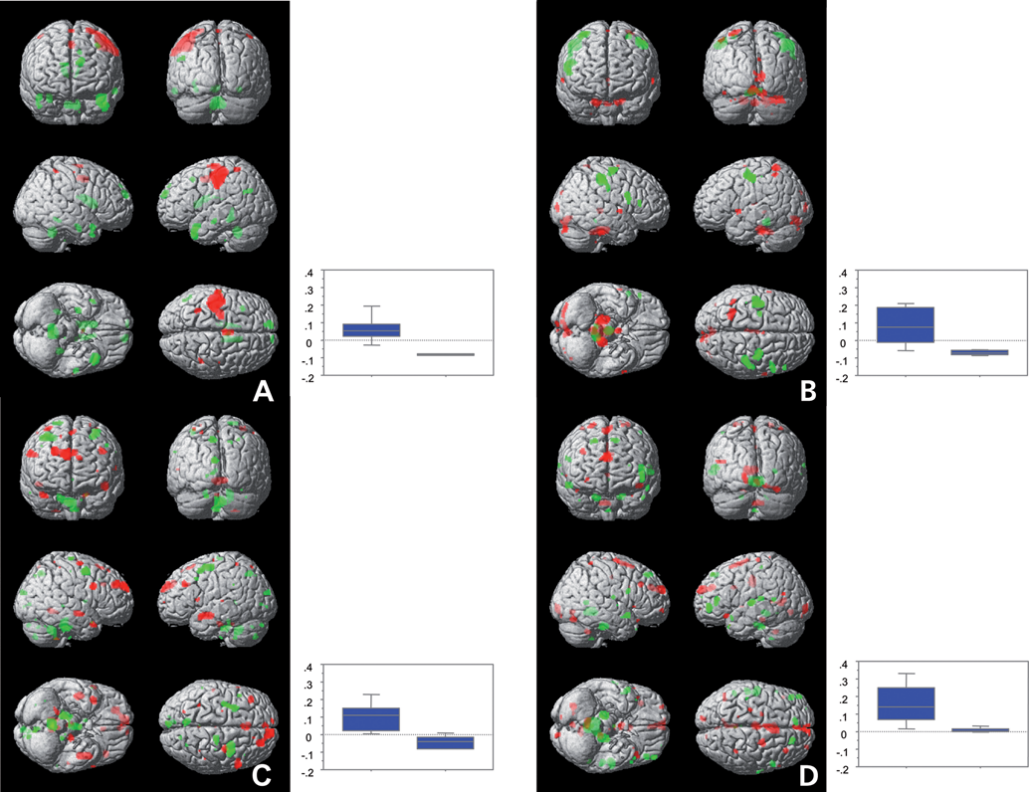
Surface renderings of the PC images 1 (A), 3 (B), 26 (C) und 32 (D) and box plots showing the associated statistical tests of the mean expression coefficients. In the surface renderings, voxels with positive loads (i.e. >99% of all voxel values) are represented in red and voxels with negative loads (i.e. <1% of all voxel values) are represented in green. The areas of the PC images are superposed on the T1-weighted MNI brain of SPM99 and shown in anterior, posterior, right and left lateral, basal and apical views. The box plots include the unpaired comparisons of P1, P2 and Ss vs. R2 (p<0.0001) (A), of P2 vs. R2 (p<0.0001) (B), of P1b vs. P2b (p<0.002) (C) and of P2a vs. R2a (p<0.005) (D). Each of the four exhibited a corrected p<0.05. The left box represents the greater expression coefficients of the first set of paradigm phases whereas the right box represents the smaller expression coefficients of the second set. The y-axis represents the normalized expression coefficients for the group comparisons, ranging from −1 to +1.

In Figure 3, the areas involved by these PCs are depicted as cortical surface renderings of the voxels lying in the first (negative load) or ninety-ninth percentile of voxel values (positive load), and the associated comparisons are represented as box plots. The involved functional and anatomical cerebral regions are summarized in Table 2.

**Table 2:** Relevant Principal Components – Involved Activation Areas.

Functional Region	Anatomical Region		*x*	*y*	*z*	Cluster Size (n voxel)	Max. Load (E = −4) *		BOLD Change **				
**PC 1 – Sensory Guided Motor Activity**													
MI, SI and premotor c., L	Pre- and postcentral g.	−40		−19	56	1024		186		+			
Superior parietal c., L	SPL	−32		−44	59	32		115		+			
Premotor c., R	Precentral g.	46		−17	57	5		17		+			
Superior parietal c., R	SPL	44		−40	57	11		112		+			
SMA, R	Medial frontal g.	2		−5	63	28		123		+			
CMA, R, L	Anterior cingulate g.	0		−2	44	116		125					
Medial prefrontal c., L	Superior frontal g.	−12		59	21	76		−47		−			
Medial prefrontal c., R	Superior frontal g.	10		63	21	16		−45					
Dorsolat. prefrontal c., L	Middle frontal g.	−34		22	43	18		−44					
Temporal pole, L	Superior temporal g.	−40		13	−21	269		−52					
Temporal pole, R	Superior temporal g.	46		11	−21	61		−44					
Angular g., L	Angular g.	−48		−63	31	30		−43		−			
Caudate nc., R	Caudate nc.	8		6	13	225		−74					
Caudate nc., L	Caudate nc.	−8		3	15	86		−62					
Hippocampus, R	Hippocampal g.	30		−11	−20	60		−46					
Fusiform g., L	Fusiform g.	−29		−34	−8	70		−45					
Fusiform g., R	Fusiform g.	28		−39	−10	30		−43					
Vermis	Vermis	0		−48	−25	359		−86					
**PC 3 – Perception of Specific Information And Short Term Memory Processes**													
Superior parietal c., L	SPL	−26		−49	63	68		120	+				
Supramarginal g., L	Supramarginal g.	−47		−49	53	16		94					
SMA, L, R	Medial frontal g.	0		−5	68	6		12	+				
STP, L	Superior temporal g.	−59		6	0	15		113	+				
STP, R	Superior temporal g.	63		−2	0	13		100	+				
Cuneus, R	Cuneus	1		−97	10	45		90					
Cuneus, R	Cuneus	10		−95	10	46		94					
Lingual g., L	Lingual g.	−2		−80	−10	36		78					
Thalamus, R	Centro-medial thalamus	4		−31	2	443		189					
Dentate nc., L	Dentate nc.	−16		−28	−22	170		264					
Dentate nc., R	Dentate nc.	18		−27	−26	239		244					
Cerebellum, R	Posterior cerebellum	36		−82	−18	48		122					
Declive, R	Vermis	−2		−83	−14	50		122					
SI, L	Precentral g.	−44		−11	48	239		−123	−				
MI, R	Precentral g.	44		−29	47	266		−94					
Ventral premotor c., R	Inferior frontal g.	53		11	22	117		−103					
Prefrontal operculum, R	Inferior frontal g.	53		28	10	36		−89					
Premotor c., L	Middle frontal g.	−27		−11	59	26		−89					
Premotor c., R	Middle frontal g.	32		−7	61	131		−115					
Reticular formation, R, L	Mesencephalon	0		−20	−6	149		−172					
Tectum, R, L	Brain stem	−8		−37	−12	208		−162					
**PC 26 – Perceived Dilemma of Indistinguishable Objects**													
Medial prefrontal c., L, R	Superior frontal g.	−8		63	19	380		118					
Dorsal premotor c., R	Superior frontal g.	34		−4	60	28		94					
Ventral premotor c., R	Superior frontal g.	57		13	27	56		96					
Dorsal prefrontal c., L	Middle frontal g.	−40		31	28	16		79					
Superior parietal c., L	SPL	−33		−48	63	6		76					
Frontoparietal operculum, L	Precentral g.	−59		12	9	5		78					
Pre-SMA und SMA, L, R	Superior frontal g.	−2		26	52	22		96					
CMA, L	Anterior cingulate g.	−4		27	28	17		80					
Retro-insular c., R	Insula	46		−8	−10	198		105					
Retro-insular c., L	Insula	−44		−8	−11	59		100					
Hippocampus, L	Hippocampal g.	−18		−20	−27	88		134					
Temporal pole, R	Superior temporal g.	39		10	−27	101		167					
Thalamus, R	Dorso-medial thalamus	4		−19	10	38		87					
Cerebellum, R	Posterior cerebellum	22		−43	−51	26		67					
Premotor c., L	Middle frontal g.	−28		−9	51	154		−192					
Premotor c., R	Middle frontal g.	32		25	50	220		−108					
Dorsal premotor c., R	Precentral g.	18		−16	67	35		−100					
Anterior cingulate., L	Anterior cingulate g.	−4		42	6	16		−96					
Superior parietal c., R	SPL	16		−74	46	5		−70					
Precuneus, L	Precuneus	−8		−62	47	62		−91					
Cuneus, L	Cuneus	−4		−90	23	40		−90					
Lingual g., L	Lingual g.	−14		−87	4	3		−85					
Thalamus, L	Postero-lateral thalamus	0		−16	−4	117		−153					
Dentate nc., L	Dentate nc.	−18		−34	−24	19		−79					
Dentate nc., R	Dentate nc.	16		−29	−27	221		−143					
**PC 32 – Explicitly Perceived Discrimination**													
Dorsal intraparietal s., R	Dorsal intraparietal s.		36	−46	65	9		79	+				
Pre-SMA und SMA, L,R	Superior frontal g.		0	9	58	109		124					
Medial prefrontal c. L,R	Medial frontal g.		2	61	19	212		156					
Dorsal premotor c., R	Precentral g.		21	−6	64	36		88					
Dorsal premotor c., L	Precentral g.		−22	−6	64	5		76					
Dorsolat. prefrontal c., R	Middle frontal g.		33	29	30	31		80					
Ventral operculum, L	Inferior frontal g.		−42	21	−13	31		104					
Intermediate cingulate, L, R	Intermediate cingulate g.		−2	13	25	31		82					
Posterior cingulate c., L	Posterior cingulate g.		−2	−14	34	182		108					
Thalamus, L ,R	Ventro-medial thalamus		2	0	0	40		98					
Medial temporal g., L	Medial temporal g.		−44	−63	17	63		78					
Cuneus, L	Cuneus		−12	−95	1	55		95					
Fusiform g., L	Fusiform g.		−4	−56	8	221		121					
Culmen, L, R	Vermis		−10	−32	−19	31		78	+				
Declive, R	Vermis		29	−71	−18	57		8					
Nodulus, R	Vermis		0	−50	−39	74		98					
SI, L	Postcentral g.		−44	−30	57	4		−79					
Lateral premotor c., R	Inferior frontal g.		55	11	22	18		−79					
Medial prefrontal c., R	Superior frontal g.		18	41	45	25		−91					
Medial prefrontal c., L	Superior frontal g.		−10	45	42	24		−90	−				
Dorsolat. prefrontal c., L	Middle frontal g.		−48	48	−4	60		−99					
Temporal operculum, L	Superior temporal g.		−52	−12	6	101		−99					
Temporal pole, R	Superior temporal g.		55	9	−11	62		−92					
Temporal pole, L	Superior temporal g.		−53	13	−16	7		−82	−				
Hippocampus, R	Hippocampal g.		28	−50	−8	16		−87	−				
Pons, R	Pons		2	−43	−50	23		−107					
Dentate nc., R	Dentate nc.		18	−26	−24	117		−191					
Dentate nc., L	Dentate nc.		−18	−24	−22	77		−176					
Vermis, L, R	Vermis		8	−39	−8	571		−207					

MI, primary motor cortex, CMA, cingular motor area; SI, primary sensory cortex; SPL, superior parietal lobule; SMA, supplementary motor area; STP, superior temporal plane; g., gyrus; c., cortex; nc., nucleus; s., sulcus; R, right; L, left.

* Max. Load  =  greatest voxel value within a cluster, representing the contribution to a defined PC.

** according to categorical comparisons; +  =  increase of blood flow, −  =  decrease of blood flow.

PC 1 discriminates between the manipulation of spheres Ss or exploration of the second objects (P2) and the following recovery (R2). The difference between exploration of the first objects (P1) and R2 exhibits a trend. In fact, the difference between exploration or manipulation of objects (P1, P2 and Ss), and R2 was significant at p<0.0001. Both active exploration of the parallelepipeds and manipulation of the spheres yielded significantly higher component expression coefficients than the recovery phases. The regions with positive loads in the left hemisphere included the sensorimotor cortex, SPL, premotor cortex, supplementary motor area (SMA), and anterior cingulate. Adjacent to the SPL, the dorsal part of intraparietal sulcus appeared bilaterally. Regions with negative loads included in the right hemisphere the caudate nucleus and the hippocampus, in the left hemisphere the superior frontal cortex and dorsolateral prefrontal cortex, and bilaterally the fusiform gyrus, and the vermis.

PC 3 discriminated between P2 and R2, i.e. exploration, comparison and decision of the second object regardless of its length relative to the first object. The expression coefficients are distinctly higher for P2 than R2. This component differs most strikingly from PC 1 in that it does not distinguish manipulation of spheres from the following intervals. The regions with positive loads include in the left hemisphere the SPL, supramarginal gyrus, lingual gyrus, bilaterally the SMA and the superior temporal gyrus, the right cuneus, the right centro-medial thalamus, and bilaterally the dentate nucleus and the right posterior hemisphere of the cerebellum. Regions with negative loads include in the right hemisphere medial frontal gyrus and motor cortex, bilaterally the premotor cortex, the tectum and reticular formation of the midbrain.

**Figure 4:**
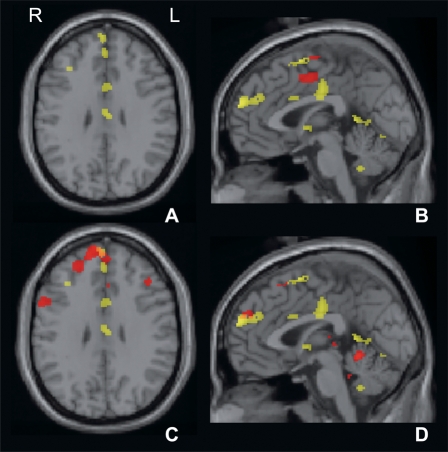
Axial (A, C) and medial sagittal slices (B, D) of the T1-weighted MNI brain with superimposed positively-loaded regions of PC 1 (red on A, B), PC 26 (red on C, D) and PC 32 (yellow on all slices). Note: (1) in the image of PC 1 the SMA and a superior dorsomedial area close to the cingulate cortex are involved exclusively; (2) in the image of PC 26 a small part of the pre-SMA and the medial-frontal cortex are implicated; and (3) in PC 32 the medial structures, i.e. the pre-SMA, the medial-frontal, the intermediate and posterior cingulate cortex, are most extensively incorporated into the network. Axial slices are at Talairach z-axis 31 mm, sagittal slices at Talairach x-axis 0.4 mm.

PC 26 distinguishes most prominently between the explorations of parallelepipeds for which the length differences are below the detection threshold, P1b and P2b. Furthermore, it discriminates with marginal significance (p<0.009 uncorrected) P1b from R2b. The component expression coefficients are distinctly higher for P1b than for P2b. The regions with positive loads include in the right hemisphere the premotor cortex, SMA and pre-SMA, anterior cingulate, and dorso-medial thalamus, the right posterior lobe of the cerebellum, and bilaterally the medial frontal and prefrontal cortex, the retro-insular cortex, and the left hippocampal gyrus. Regions with negative loads include bilaterally the premotor cortex, the left precuneus and cuneus, the left postero-lateral thalamus and bilaterally the dentate nucleus (see also Fig. 4).

PC 32 involves the presentation of the second parallelepiped in the case that the difference in lengths of the pair exceeded the discrimination threshold (P2a). The most significant discrimination is that between P2a and R2a; marginally significant (p<0.009 uncorrected) were discriminations between P2a and P2b or Ss. The component expression coefficients are distinctly greater for P2a than for R2a, P2b or Ss, respectively. The regions with positive loads include in the right hemisphere the dorsal part of the intraparietal sulcus, the dorsolateral prefrontal cortex, bilaterally the pre-SMA and SMA, the medial prefrontal cortex, the premotor cortex, the intermediate and posterior cingulate, in the left hemisphere the cuneus, the fusiform gyrus, and vermis. Regions with negative loads include in the left hemisphere the postcentral gyrus, the dorsolateral prefrontal cortex and the temporal operculum, in the right hemisphere the hippocampus, bilaterally the superior frontal gyrus and the temporal pole, and the right pons and the vermis (see also Fig. 4).

### Categorical analysis

Categorical comparisons corresponding to the differences between the phases and conditions of the paradigm identified as relevant by the PCA were calculated. Shown in Table 3, the subject-condition images of the categorical contrasts correspond to the subject-condition expression coefficients evaluated using the t-tests in Table 1. The significant BOLD changes of the categorical contrasts are included in Table 2 in order to further facilitate comparison with PCA. If there were multiple candidates, the best fit with the corresponding PC according to the correlation of voxel values was selected.

**Table 3:** Categorical analysis of activation maxima (SPM99).

Categorical comparisons			Principal components			
			**1**	**3**	**26**	**32**
P1+P2+Ss	vs	R2	0.70			
P2	vs	R2		0.32		
P1b	vs	P2b			0.01	
P2a	vs	P2b				0.34

Display of the conditions used in the t-tests constituting the categorical comparisons and the analogous t-tests of subject-condition component expression coefficients; unpaired t-tests were used in the first two categorical comparisons and paired t-test in the third and fourth. For selection criteria of the categorical comparisons, see *Results*, *Categorical comparisons*. The correlation coefficients (r) of voxel values between PCA and the related categorical comparisons are indicated.

Corresponding to PC 1 was the categorical comparison of P1, P2 and Ss with R2, independent of whether the object pairs could be discriminated; i.e. we pooled contrast corresponding to t-test 1–4 and 9 of the Table 1. The comparison reveals an extended region of bilaterally increased rCBF that is concentrated on the left and includes the primary motor cortex, the primary somatosensory cortex, the premotor cortex, the SMA, the posterior parietal lobe with the SPL and the adjacent intraparietal sulcus. Additional increases of blood flow appear in the premotor cortex, the anterior cingulate cortex, the insular cortex, and the putamen bilaterally, in the left red nucleus, and in the right anterior cerebellum. Significant decreases of flow comprise regions of the left cerebral hemisphere including the inferior parietal lobule with supramarginal gyrus and angular gyrus, the lateral occipital gyrus, the posterior cingulate cortex, and the middle temporal gyrus proximate to the temporal pole. Determined to be 0.7, the coefficient of voxel-value correlation between the t-statistical map of the categorical comparison and the component image of PC 1 indicated a notable correlation.

Corresponding to PC 3 was the categorical comparison of P2 with R2, regardless of whether the object pairs could be discriminated. The pattern of significant blood flow increases and decreases was similar to that of the categorical comparison of P1, P2 and Ss with R2. Differences in the pattern of blood flow increases occur in the sensorimotor cortex, which involves in this second comparison more of the right hemisphere, including the parietal operculum. The pattern of flow decreases is less extended in this comparison, especially in the lateral occipital gyrus, in the left temporal lobe, and in the left posterior cerebellum. The coefficient of voxel-value correlation, 0.32, indicated moderate correlation between this categorical comparison and PC 3.

Corresponding to PC 26 was the categorical comparison discriminating the presentations of pairs which could not be distinguished, P1b and P2b. This comparison showed a circumscribed signal increase during P2b, which was localized in the primary motor and primary somatosensory cortex on the left side. The pattern of significant blood flow changes differed from the categorical comparison of P1, P2 and Ss with R2 in that it did not involve premotor, cingulate or parietal areas. The coefficient of voxel-value correlation between this categorical comparison and PC 26 was negligible.

Corresponding to PC 32 was the categorical comparison P2a with P2b. The direct comparison of distinguishable and indistinguishable objects during this phase is of primary interest, as it reflected the explicit somatosensory discrimination, the primary aim of this investigation. The patterns of significant blood flow increases and decreases are markedly different from those of the original categorical comparison of P1, P2 and Ss with R2. Significantly increased blood flow appeared in three compact regions comprising the dorsal part of intraparietal sulcus of the right hemisphere, the ventral premotor cortex of the left and the right vermis. Significant decreases included several areas in the middle and superior temporal gyri, the dorsolateral prefrontal cortex, putamen, and the ventral visual cortex of the left hemisphere, the parahippocampal gyrus, precuneus and posterior cingulate cortex of the right hemisphere, and the lateral occipital gyrus bilaterally. The coefficient of voxel-value correlation, 0.34, between the t-statistical map of the categorical comparison and the component image of PC 32 indicated moderate correlation.

## Discussion

In this study we used PCA to identify the neural networks engaged in tactile discrimination of rectangular objects. We applied this method, since tactile object discrimination is a psychologically complex endeavor even if performed under well defined experimental conditions as in this experiment. Before we discuss our findings we would like to emphasize that PCA is a data driven method explaining the variance in the image data. We supplemented this analysis in a second step by inferential testing in which we asked which PC differentiated the experimental conditions across the subjects. This was similar to a categorical comparison of the BOLD signal changes related to the task conditions. In fact, for each contrast we identified a PC. Since the mean contrast in pattern expression was statistically significant for each component identified, it provided independent evidence of the component's functional relevance. Thus, while the categorical comparison of the hemodynamic changes reduced the data to the task specific BOLD signal changes, our PCA approach showed the distributed neural network including brain areas in which BOLD signal changes were not significant according to the categorical comparison. Moreover, image volume correlations between categorical comparison and PC images suggest that changing the threshold for “significant” regional activity would not dramatically alter the apparent dissimilarity between PCA and SPM contrast patterns. Consequently, the interpretation of the observed neural patterns was guided mainly by the task conditions. In addition, the anatomical structures involved in the PCA allowed to tentatively suggest a physiological implication - an approach similarly applied in categorical comparisons as well.

We were able to distinguish four PCs corresponding to neural networks implicated in somatosensory discrimination. Showing significant differences among stimulation phases and shape characteristics of the object pairs, they ranked 1st, 3rd, 26th and 32nd among the PCs according to the percentage of variance explained.

The formulation of the statistical tests indicates the following interpretations: PC 1 reflects sensory-guided motor activity, PC 3 relates to the perception of shape characteristics and short term memory function, PC 26 to the awareness that the length difference could not be discriminated, and PC 32 to the recognition that the length difference could be discriminated (Table 3). These principal components therefore represent a hierarchy of neural networks corresponding to a partitioning of the two-alternative forced-choice stimulation paradigm. Among the cerebral regions evident in the principal components are the left posterior parietal lobe and premotor cortex in PC 1, the left SPL and the right cuneus in PC 3, the medial frontal and orbitofrontal cortex bilaterally in PC 26, and the right intraparietal sulcus, anterior SPL and dorsolateral prefrontal cortex in PC 32. The corresponding categorical comparisons showed only partial involvement of these crucial regions. Our observations suggest the following interpretations.

### Principal Component 1

This PC distinguishes between phases of tactile exploration associated with haptic information processing (parallelepipeds) or pure manipulation (spheres) and the corresponding pauses. The component therefore represents the concerted, directed and adaptive motion of the fingers which constitutes the basis of both actions.

In the principal component image, the sensory and motor cortices contralateral to the exploring hand appear prominently with large positive loads. Only the SPL appears distinctly bilateral in the network, although minimal involvement of the premotor cortex ipsilateral to the exploring hand is indicated. The SPL, the dorsolateral premotor cortex and parts of intraparietal sulcus are essential nodes of a frontoparietal network regulating the manipulation of objects [5]. It should be mentioned, that the activation in the anterior SPL which is adjacent to but spatially distinct from the anterior intraparietal sulcus (AIP), has been shown to mediate finger aperture for object grasping [24]. Particularly interesting is the involvement of medial surface regions of both hemispheres, i.e. the SMA and the left superior dorsomedial area close to the cingulate cortex. Similar activation patterns have been found in experiments in which macaque monkeys performed remembered sequences of grasping motions in response to reward [25]. Activation studies of humans have demonstrated the significance of the anterior cingulate adjacent to the pre-SMA for valuation of sensations, mental activity, motor imagery, and attention to an upcoming action [26]–[31]. However, a lesion study employing four test paradigms did not confirm the findings of the activation studies [32]. This discrepancy motivates the speculation that these regions modulate a motor function in relaying the salience associated with an activity, but are not essential for its execution.

The pattern includes regions of the dorsal medial cortex bilaterally and of the left dorsomedial prefrontal cortex which may mediate self-reflection, i.e. introspection and directed attention in contrast to external perception [33]. Additional constituents are areas of the temporal-parietal-occipital cortex that effect tasks such as collection and retrieval of visual patterns, directed attention to visual stimuli and pattern discrimination [34]. The negative sign of the loads indicates that this pattern is inversely correlated in the PC with the areas characterized by positive loads.

### Principal component 3

This PC distinguishes between presentation of the second parallelepipeds and the recovery phase regardless of whether the length difference exceeds or is below the discrimination threshold. The component appears to represent the perception of shape characteristics as well as the involvement of short-term memory required for comparison of the objects and discrimination.

Consistent with this hypothesis is the appearance with large positive loads of the posterior parietal lobe contralateral to the exploring hand, including the SPL and the adjacent intraparietal sulcus and supramarginal gyrus. This is in accordance with recent observations [35], [36]. A functional differentiation between the SPL and the intraparietal sulcus has been pointed out recently [37]. This study implicated the SPL in the processing of spatial coordinates to which attention is primarily directed, while claiming that the horizontal segment of the intraparietal sulcus mediated changes in stimulus configuration. Applied to the tactile discrimination paradigm, the intraparietal sulcus seems to monitor the change of kinesthetic object properties upon presentation of the second object. Additional nodes in the visual association cortex indicate the transformation of somatosensory into visual cues, proceeding most likely via Brodmann area 5 as a stage of form recognition [38]. The appearance of the cuneus in the component image reflects specific data to be deposited in episodic memory [39]. The remaining nodes of the network in the dentate nucleus and the right posterior cerebellum probably indicate attention and sensory learning [40]. The bilateral occurrence of the premotor and motor cortices with small negative loads in the network underscores the perceptual significance of this principal component.

### Principal component 26

This PC distinguishes between presentations of the first and second parallelepiped that could not be discriminated. The occurrence of this PC indicates the particular condition of being confronted with indistinguishable objects, which presumably places special demands on motivational control [41].

The PC image is characterized particularly by the marked involvement of the medial prefrontal cortex. Behavioral studies of patients with lesions of this region have indicated its importance for the subjective control of behavior [42]. The neighboring anterior cingulate cortex, also a constituent of this PC image pattern, contributes to behavioral control through its function in the recognition of errors and monitoring of conflicts [43], a function that would be stimulated by a paradigm that requires the discrimination of indistinguishable objects. The involvement of the medial thalamus indicates the activation of the afferent dopaminergic mesocortico-limbic system [44]. In view of its anatomical connections with other regions of the limbic system, the rostral anterior cingulate cortex probably mediates between motivation and action [45], [46], a conjecture supported by studies showing activation of this region by both externally directed attention and emotional stimuli [47]. Internally initiated motor activity is an additional feature of this network. Critical nodes for self-generated motor activity, stimulated by subjective states, are presumably the pre-SMA found in the PC image, an essential structure in higher order motor control [48]–[50]. Other activation studies indicate that the pre-SMA is the most likely source of the readiness potential [51], [52].

The PC image further indicates participation of the dorsolateral and medial prefrontal cortices suggesting the simultaneous processing of external stimuli produced by the condition. Reciprocal activations of these two regions have been described in the literature according to the content and relationship of internal or external stimuli [53]. In summary, this principal component suggests a critical state in the discrimination of indistinguishable objects that begins with high motivation as suggested by the greater expression coefficients of the first presentation, but ends with demotivation after the subject has definitely perceived his dilemma.

### Principal component 32

This PC attains significance in the t-test discriminating between the presentation of the second parallelepiped of a distinguishable pair and the following recovery. It therefore describes the explicit recognition of distinguishable cues which constitutes the basis of somatosensory discrimination, and the decision [21]. The identification of a significant network pattern demonstrating cognitive activity during the presentations of the second object is noteworthy. Involving not only tactile exploration and memorization but also on-line comparison in working-memory and decision, it reflects the more intense haptic information processing during this phase than during the presentations of the first object [54]. Consistent with the involvement in haptic information processing, the PC image shows the ipsilateral intraparietal sulcus with its dorsal part embedded in a network including the dorsal premotor and the dorsolateral prefrontal cortices of the right hemisphere. The participation of the premotor cortex reflects the sensorimotor processing required by the adaptive grasping and manipulation of the object [1], [7], [8]. The dorsolateral prefrontal cortex is essential for the decoding of object features and, during the exploration of the second parallelepiped, for the retrieval of features describing the first parallelepiped necessary for the comparison [55], [56]. The implication of the prefrontal cortices in the network may indicate sustained directed attention to the task, which contrasts the attenuated BOLD signal in familiar tasks after practice [57].

The basic ability tested by the paradigm, the recognition of the changing stimulus configuration, i.e. kinesthetic impulses in the transition from the first to the second parallelepiped, was recently classified as a specific function of the horizontal segment of the intraparietal sulcus [37]. The importance of this region during the somatosensory discrimination of objects differing in their 3D structures has also been pointed out previously by Bodegard et al. [58]. The anatomical and functional classifications of the intraparietal sulcus are based on historical cytoarchitectonic studies, on animal experiments and morphological classifications in macaques, and on functional imaging studies of humans with fMRI and PET [24]. A recent probabilistic cytoarchitectonic map has identified two regions in the anterior wall of the intraparietal sulcus in humans [59]. The human counterpart of the AIP has been suggested to lie deep in the horizontal course of the intraparietal sulcus, and appears to correspond to the region IP2 identified by Choi et al. [59]. However, the activation field delimited in our study lies more dorsally within the intraparietal sulcus in correspondence to activation studies exploring gesture processing [60] or cyclic flexion-extension movements of the wrist [61]. We suggest that this area of activation in the dorsal part of the intraparietal sulcus plays a key role for the recognition of subtle differences of kinesthetic information extracted from different objects, i.e. the defined objective of the study task. Given that exploratory finger movements evolve automatically and are tightly scaled to the features of the objects [62], they sample both information about the object surface and simultaneously kinesthetic information about the object shape [5], [35]. The activation along the dorsal intraparietal sulcus we found is probably due to an enhanced processing demand related to the discrepancy of the greater and changing kinesthetic signal in the presence of an identical signal related to the surface characteristics.

Parallel to activity in nodes processing object-related impulses is activity in key cerebral regions responsible for subjective assessment and validation: multiple medial regions of the cerebral hemispheres including the pre-SMA, the anterior, mid and perigenual cingulate cortices and medial prefrontal cortex [62]. Of particular interest is the involvement of the pre-SMA, which Lau et al. have shown to play a key role in making decisions (“Go versus No-go”) [40]. These areas appear to be activated in expectation of receiving decisive data during the presentation of the second object, effecting increased salience of the resultant impulses and producing a form of reward [40]. Comparison of component images of PC 26 and 32 show that this system is more extensively involved in the presence of distinguishable objects. The involvement of the mid and posterior cingulate cortex can probably be attributed to their monitoring function [64].

### Conclusions

Principal component analysis produces without a priori assumptions component images covarying independently and implying the functional connectivity of the constituent regions [10]–[12]. Applied to the fMRI study of tactile exploration, principal component analysis provides a means of revealing neural networks recruited by specific phases of the stimulation paradigm as inferred a posteriori using statistical inference. Thus, the four principal component images that discriminate most clearly among the phases and conditions of the paradigm show the nodes of the networks mediating somatosensory discrimination.

The component images PC 1, 3 26 and 32 reveal distinctly different aspects of the paradigm, consistent with the finding that decisions of the kind required by the tactile exploration paradigm are a consequence of independent processes [65]. The involvement of the anterior intraparietal sulcus and the superior parietal lobule during object exploration and the associated cognitive processing manifests clearly their role in tactile exploration and form recognition. The participation of the medial frontal cortex in exploration and processing in PC 26 and PC 32 is of particular interest. Moreover, the cingulate cortex appears to express bilaterally the assessment and validation of perceived stimuli. The emergence of the dorsal intraparietal sulcus in the right hemisphere of PC 32 contrasts to its appearance in the left hemisphere in PC 1, indicating the role of the right hemisphere in explicit information processing and recognition [66], [67]. In relation to directed attention, the ipsilateral dorsolateral prefrontal cortex probably serves the decoding and retrieval of stored object features from working memory [55]. Finally, the pre-SMA may mediate the Go/No-Go function as part of the cerebral pattern related to the decision based on the somatosensory percept [40].

The statistical maps of the categorical comparisons, especially those based on rest, reveal only regions that serve the acquisition of sensorimotor information, but not those that are suggested to mediate its valuation, in particular areas like the medial prefrontal cortex, the cingulate and paracingulate cortex as represented in the medial sagittal slices of Fig. 4 [68]. Accordingly, only the sensorimotor pattern of the first categorical comparison evidenced a substantial correlation with PC 1 and, additionally, neither the earlier published SPM analysis of tactile object discrimination [58] nor the categorical comparisons of this study yielded neural regions not seen in one of the PCs. Thus, activation of some network nodes, specifically those involved in the regulation of sensory information processing, may appear in salient PCs but not attain the critical significance threshold in categorical comparisons [17]. In sum, the differentiated patterns of the independent components and the involvement of regions not revealed by statistical parametric contrast maps demonstrate the power of principal component analysis as a useful complement to classical categorical analysis. Further developments of multivariate analysis techniques may help to refine our understanding of the connectivity between multiple brain regions and their relationship to human behavior [70].
